# National smokefree law in New Zealand improves air quality inside bars, pubs and restaurants

**DOI:** 10.1186/1471-2458-7-85

**Published:** 2007-05-18

**Authors:** Nick Wilson, Richard Edwards, Anthony Maher, Jenny Näthe, Rafed Jalali

**Affiliations:** 1 Department of Public Health, University of Otago (Wellington), Wellington, New Zealand.; 2 Medizinische Fakultät der Charité – Universitätsmedizin Berlin, Berlin, Germany.

## Abstract

**Background:**

We aimed to: (i) assess compliance with a new smokefree law in a range of hospitality settings; and (ii) to assess the impact of the new law by measuring air quality and making comparisons with air quality in outdoor smoking areas and with international data from hospitality settings.

**Methods:**

We included 34 pubs, restaurants and bars, 10 transportation settings, nine other indoor settings, six outdoor smoking areas of bars and restaurants, and six other outdoor settings. These were selected using a mix of random, convenience and purposeful sampling. The number of lit cigarettes among occupants at defined time points in each venue was observed and a portable real-time aerosol monitor was used to measure fine particulate levels (PM_2.5_).

**Results:**

No smoking was observed during the data collection periods among over 3785 people present in the indoor venues, nor in any of the transportation settings. The levels of fine particulates were relatively low inside the bars, pubs and restaurants in the urban and rural settings (mean 30-minute level = 16 μg/m^3 ^for 34 venues; range of mean levels for each category: 13 μg/m^3 ^to 22 μg/m^3^). The results for other smokefree indoor settings (shops, offices etc) and for smokefree transportation settings (eg, buses, trains, etc) were even lower. However, some "outdoor" smoking areas attached to bars/restaurants had high levels of fine particulates, especially those that were partly enclosed (eg, up to a 30-minute mean value of 182 μg/m^3 ^and a peak of maximum value of 284 μg/m^3^). The latter are far above WHO guideline levels for 24-hour exposure (ie, 25μg/m^3^).

**Conclusion:**

There was very high compliance with the new national smokefree law and this was also reflected by the relatively good indoor air quality in hospitality settings (compared to the "outdoor" smoking areas and the comparable settings in countries that permit indoor smoking). Nevertheless, adopting enhanced regulations (as used in various US and Canadian jurisdictions) may be needed to address hazardous air quality in relatively enclosed "outdoor" smoking areas.

## Background

There is growing international interest in the use of smokefree legislation for improving air quality and protecting the health of workers and the public. Comprehensive smokefree laws have been introduced in such jurisdictions as Ireland, Italy, Malta, New Zealand, Norway, Scotland, Sweden and various US states (eg, New York and California). Many other jurisdictions are in the process of enacting such laws, including coverage of bars and restaurants.

In New Zealand, the *Smoke-free Environments Act *that was passed in 1990 made many indoor workplaces smokefree (including: shops, most offices and some other workplaces along with partial restrictions on smoking in cafés and restaurants). In December 2004, nearly all the provisions of a new *Smokefree Environments Amendment Act *of 2003 came into force. This new smokefree law had the effect of making all bars/pubs and restaurants completely smokefree, along with nearly all other workplaces and associated facilities not covered by the 1990 Act (eg, warehouses, factories and lunchrooms).

There are limited published data on the impact of the Smoke-free Environment Act (1990) [1] and also on the new law that became operational in December 2004 [2]. In terms of quantifiable aspects of air quality, there has been just one study which assessed biomarkers of secondhand smoke (SHS) exposure before and after the 2003 amendment [3]. It found that changes in salivary cotinine levels among volunteers entering bars were substantially less after the new law (representing a 90% reduction in SHS exposure).

In this study we aimed to: (i) assess compliance with the new smokefree law in a range of hospitality settings 18 months after the law came into force; and (ii) to assess the impact of the new law by measuring air quality and making comparisons with air quality in outdoor smoking areas and with international data from hospitality settings.

## Methods

The methodology of this study followed the processes of the international "Global Air Monitoring Study" being coordinated by the Roswell Park Cancer Institute in New York [4]. However, it included a number of additional methodological features including the more structured sampling of pubs, bars and restaurants.

### Selection of urban pubs and restaurants

Our initial sampling method involved the random selection of bars and restaurants from the Central Business District (CBD) of Wellington City. This is an area based, with simplifications for some boundaries, on the official "Central Area Boundary" of the Wellington City Council [5]. This sample frame was also used for other settings detailed below (with the exceptions being for the hospital, the airport and for some bus and taxi routes that extended beyond the CBD). We aimed to sample equal numbers of bars and restaurants with and without linked "outdoor" smoking areas. Our sampling ceased once we had four venues in each of the four different categories. The selection of map coordinates of the CBD was based on random number tables. These tables also determined the direction to take on foot from the selected map coordinate. The first bar or restaurant encountered was then assessed and sampled. Bars were defined as venues that had any "bar area" that served alcohol and with selling alcohol being considered to be the main purpose of the establishment. Restaurants were defined as venues having sit-down meals with menus and with selling food being considered to be the main purpose of the establishment. Data collection occurred on Friday and Saturday nights in the 5 pm to 10 pm time slot during May/June 2006 (late autumn and early winter in New Zealand). To facilitate outdoor sampling of adjacent "outdoor" smoking areas we selected days when it was not raining.

### Additional selection of urban "bars"

After field experience with the sampling method detailed above, we also undertook another method that was more orientated towards sampling more traditional "bars" where the focus was on serving drinks rather than food. This was because we suspected that these venues may be more likely to have clientele with higher smoking prevalence rates, and hence infringements of the law may be more likely in these venues. The selection process involved searching the electronic yellow pages (Telecom, 2006) under the categories of "bars & brasseries" and "hotels & taverns" and within the category of "Wellington CBD". From these entries (n = 48+88 respectively) we selected those bars that met the following criteria: (i) the word "bar" was in the name; (ii) there were none of the following additional words in the listed name of the bar: "café", "restaurant", "grill" or "pool saloon"; (iii) the bar was not also listed under the "adult entertainment" category of the yellow pages. These steps generated a list of 14 bars from which we randomly selected 11 using random number tables. Data collection occurred on one day, Saturday 24 June 2006 (from 2 pm to midnight). By chance, none of the sampled bars had adjacent "outdoor" smoking areas (ie, no "sit down" outside tables).

### Selection of small town and rural pubs

The South Wairarapa area was selected as a convenience sample of a rural area (to minimise travel time and travel costs from Wellington). A search of the "Yellow Pages" Directory found 10 entries for "Hotels & Taverns" in "Featherston & Districts" and five entries in "Carterton". From these entries a purposeful sample of the more remote and more traditional pubs was made ie, those that focused on serving drinks rather than food. This selection was based on website information and included those venues with the words "tavern" or "hotel" or "inn" in their name (since these terms tend to reflect more traditional establishments rather than more tourist-orientated or "up-market" establishments). The nine selected pubs were in small towns (n = 6) and in more remote rural settings (n = 3). All these pubs were visited on a single day (Friday, 16 June 2006) starting at midday.

### Sampling of other indoor and outdoor settings

We also used convenience sampling in Wellington city (mainly in the CBD) to investigate a range of ten other smokefree venues, including: cafés, shops, offices, a hospital, a smokefree pedestrian arcade; and nine transport settings (buses, a taxi, a train, bus and train stations, and an airport). Finally we identified six outdoor settings in the Wellington CBD (ie, walkways, roadside areas, and a city park) that were not designated smokefree except for one of the walkways.

### Sampling of relatively enclosed "outdoor" smoking areas

To allow for comparisons with indoor areas, we also included outdoor smoking areas where these were available at the included venues. This was provided there was at least one occupant present other than the investigator and at least two cigarettes were smoked during the data collection period. Four of the these outdoor areas were from the 34 included pubs, bars and restaurants, while two others were purposively included as they were known to be relatively enclosed (ie, with walls on at least three sides).

### Air quality data collection

As per the processes of the international "Global Air Monitoring Study", indoor venues were visited unannounced by either one or two of the investigators. Also to avoid affecting occupants' behaviour, we behaved as normal customers (ie, bought drinks or food in the pubs and restaurants). At each indoor sampling site, the busiest room in the pub or restaurant was identified and an attempt was made to obtain central seating (though in some busy settings this was not always possible). We ensured that the sampling was not within two metres of any open doors or windows leading to the outside or kitchen areas. For "outdoor" smoking areas we aimed to sample from the middle of the area and not in the immediate vicinity (< 1 metre) of anyone smoking.

Data were collected on fine particulates which are defined as being 2.5 μm in diameter or less (ie, "PM2.5"). The data were collected using a TSI SidePak AM510 (TSI, Inc, St Paul, USA) portable real-time air quality monitor (a photo of which is at: [6]). This device recorded average levels of these particulates (PM_2.5_) over one minute periods. The use of the monitor followed a protocol modified from one developed for a US study [7] and as used in a previous UK study by one of the authors (RE) [8]. This monitor was zero-calibrated prior to use and was fitted with a 2.5 μm impactor with an air flow rate of 1.7 l/min. The air flow rate has been validated in the New Zealand setting using a pneumotachograph (Hans Rudolph 4813 pneumotachograph, Vacuumed differential pressure transducer 4500, Vacumetrics, California, USA), and was within 10% of the stated flow rate.

A length of Tygon™ tubing was attached to the inlet of the *SidePak*, with the other end left protruding (slightly) outside the bag. The bag with the sampling equipment was carried or placed on a seat or table wherever possible to sample the ambient air. Recording occurred for 35 minutes (to ensure a 30 minute sample). Where indoor sampling occurred in hospitality settings, we also sampled for at least 5 minutes from directly outside the venue (but 5 metres away from any people smoking on the street).

### Observation data collection

At each indoor setting, additional information was systematically collected on a preformatted data collection sheet. This included recording number of people in room/area (at 0, 15 and 30 minute intervals) and the number of lit cigarettes (at 0, 15 and 30 minute intervals). A sketch was drawn of the layout of the room and the location of the sampling site in relation to doors and to any "outdoor" smoking areas.

### Data analysis

A calibration factor of 0.32 was applied to the measured data based on calibration work with a ThermoMIE personalDataRAM model pDR-1200 real-time aerosol monitor (ThermoAndersen, Inc, Smyrna, GA, USA) and as used in other studies using the *SidePak *monitor [7-10]. This type of monitor has also been calibrated against standard pump-and-filter gravimetric methods [10]. The recorded measurements were downloaded to a personal computer for analysis using TRAKPRO version 3.4 and EpiInfo (CDC Atlanta). Mean time-weighted averages and peak levels for each setting were calculated.

### Ethics approval

Ethics approval was obtained through the University of Otago's ethical review system (Category B approval). At the international level the "Global Air Monitoring Study" has obtained ethical approval from the Roswell Park Cancer Institute Ethics Review Board.

## Results

### Observational data on compliance

In the 34 bars, pubs and restaurants there were 3038 people (including staff) observed to be present indoors in the venue at the three observation times (though many were the same individuals observed at different time points). None of these people were observed smoking indoors. Outside the official observation period as the investigators were leaving, one person was observed smoking inside one of the rural pubs. Similarly, of 747 people observed during the three observation time points inside other legally smokefree indoor settings, none were smoking. No smoking was observed inside any of the vehicles in which sampling occurred (buses, trains or taxis).

### Air quality data

Air quality data collection was successful at all indoor hospitality sites except for one rural pub where the data was not properly stored on the monitor and for one urban bar where the data was invalidated by the use of a smoke machine used to generate "atmospheric effects" (ie, the results exceeded the machine's measurement limits). Summary data from the various sampling sites are shown in Table 1 (with more detailed venue-specific data in the Appendix). The bars/pubs and restaurants had relatively low levels of PM_2.5_, in the range of 13 to 22 μg/m^3^. The figures for other smokefree indoor settings and smokefree transportation settings were somewhat lower (Table 1).

**Table 1:** Fine particulate levels at various sites in Wellington and Wairarapa in 2006 (for recorded one minute sampling over 30-minute periods in each venue for PM_2.5 _in μg/m^3^)*

**Type of venue (n)**	**Details (sampling strategy)**	**Mean (SD)**	**Median**	**Mini-mum**	**Maxi-mum**
Bar area (n = 8)	Wellington Central Business District (CBD) (random selection)	22 (7)	19	10	56
Bars – more traditional (10)	Wellington CBD (telephone directory)	13 (11)	8	2	94
Restaurants (8)	Wellington CBD (random selection)	14 (7)	13	2	37
Rural pubs (8)	Wairarapa (telephone directory for more traditional)	17 (21)	10	1	109
Outside of all the pubs/bars/restaurants (34) *	Central Wellington & Wairarapa (as details in the first four rows of this table)	14 (14)	7	0	137
Smoking areas of bars/pubs/restaurants (4)**	Wellington CBD (random selection)	36 (27)	19	7	189
Relatively enclosed smoking areas attached to bars (2)#	Wellington CBD (purposeful sampling)	124 (83)	116	20	284
Transportation settings (10) *	Wellington (convenience sample). Includes: buses (5), taxi, train, bus station, train station and airport.	13 (8)	11	1	62
Other indoor settings (9)	Wellington (convenience sample). Includes: cafés (2), offices (2), hospital, library, club, shopping centre, and supermarket.	3 (2)	2	0	14
Other outside (6)	Central Wellington (convenience sample). Includes: park (2), walkway (2), and roadside (2).	7 (4)	6	2	50

* The sampling outside venues was for only five minutes and for some transport settings it varied by the length of the trip. But all the other results in this table are for 30 minute sampling periods.

** For those "outdoor" smoking areas that were fully occupied by at least one person (beside the investigator/s) throughout the 30 minute period and which had a least two cigarettes smoked during this time.

# In two additional bars the smoking areas were not occupied at the survey time, and so these results are not included in this category.

SD – Standard deviation.

Considering all the bars, pubs and restaurants together (n = 34), the mean level of PM_2.5 _was 16 μg/m^3 ^and median was 14 μg/m^3^. The levels directly outside these venues were slightly lower at 14 and 8 μg/m^3 ^respectively (though this was not a statistically significant difference: Kruskal-Wallis test, p = 0.18).

The highest indoor levels observed were at a rural pub where a peak of 109 μg/m^3 ^and mean levels of 66 μg/m^3 ^outside and 63 μg/m^3 ^inside were measured. The high particulate levels may have been due to a rubbish fire that was observed burning just outside this pub, as well as the presence of a lit open fire indoors.

**Figure 1:**
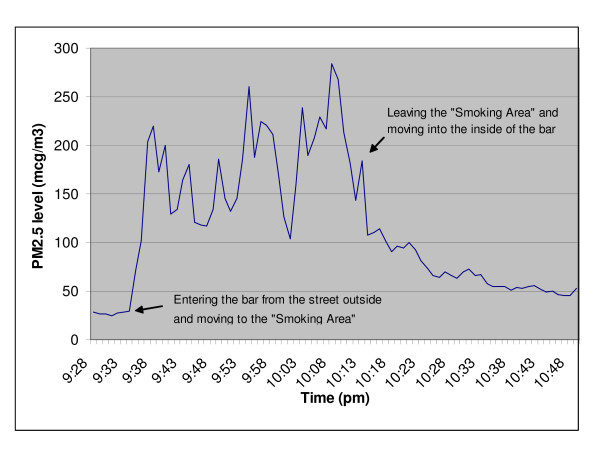
Fine particulate levels (PM_2.5_, μg/m^3^) at a Wellington bar/restaurant – outside the venue, in the "outdoor" smoking area, and then indoors (a non-smoking area). This bar/restaurant was purposefully selected as it had a semi-enclosed "outdoor" smoking area that was unlikely to be substantially influenced by ambient wind and had a high number of cigarettes being smoked at the three observation time points (mean = 11, range = 6–20). Indoor air quality was relatively poor in this venue compared to the mean results in Table 1. This may have been due to SHS movement into the indoor area, high candle numbers and the open connection to a kitchen area.

The mean PM_2.5 _level for the four randomly selected "outdoor" smoking areas was 36μg/m^3 ^(Table 1). These areas had an average of four cigarettes burning at each of the three observation time points. Within these venues the smoking area with the highest mean value (75 μg/m^3^) and highest maximum value (189 μg/m^3^) was the one that was most enclosed (ie, with four walls and a partial roof). The other three smoking areas were far more exposed to the open air and wind (two were on balconies and one on the footpath outside the entrance). Particulate levels were especially high in the two purposefully sampled relatively enclosed "outdoor" smoking areas (Table 1). The area with the highest levels had a 30-minute mean of 182 μg/m^3 ^and a maximum of 284 μg/m^3^(Figure 1). The fine particulate level in this smoking area was around six times higher than the outdoor air, and over three times higher than in the non-smoking area indoors.

## Discussion

### Observational data on compliance

These data indicate that smoking in legally smokefree settings, including hospitality settings, is extremely rare and that the smokefree law is being complied with. This finding is consistent with a national survey of 193 bars [11] where smoking was observed in only five (3%) of bars and among less than 1 in 400 of the patrons, and a three city survey that detected one person smoking out of 9610 bar customers [3]. It is also consistent with the rarity of prosecutions for breaches of the most recent smokefree law and the high levels of popularity by the public and even smokers themselves for the new law [12].

### Air quality data

The low levels of fine particulates seen in this study also indicate that there is good compliance with the smokefree law. The finding that indoor particulate levels in hospitality settings still tended to be higher than other indoor settings (shops, offices etc) and the environment immediately outside is not surprising. It is likely to be because hospitality settings have multiple sources of fine particulates including: cooking [13], open fires [14], unflued gas heaters, candles, and SHS drifting from outside through doorways, windows and ventilation inlets.

New Zealand has no legal limits for PM_2.5 _levels and this is only the third New Zealand published study that reports levels of PM_2.5. _One of these related PM_2.5 _levels inside a car with smoking [15], and another reported outdoor levels for Auckland [16]. The latter found similar levels (ie, mean = 11 μg/m^3^; range = 2–38, for 24-hour monitoring) to the mean of 14 μg/m^3 ^for outside hospitality settings found in this study.

### Health hazard

The World Health Organization (WHO) guidelines for 24-hour mean PM_2.5 _levels are 25μg/m^3 ^[17]. The mean fine particulate levels in indoor hospitality settings in New Zealand are now well below the WHO's 24-hour guideline level but in the "outdoor" smoking areas are above it. Indeed, the mean level for one such area (at 124 μg/m^3^, Table 1) would result in workers exceeding this daily exposure limit in just over four hours of work (if taking the conservative assumption of exposure levels in all non-work settings being smokefree and being only 5 μg/m^3 ^in the rest of a 24-hour day).

### International comparisons

Air quality sampling results from different studies are shown in Table 2. These indicate that the findings for New Zealand hospitality settings in this study are fairly similar to those in other jurisdictions with similarly comprehensive smokefree laws. Also of note are that PM_2.5 _levels for venues where smoking is (or was) permitted can be very high and are similar to the findings for the semi-enclosed "outdoor" smoking areas in New Zealand.

**Table 2:** Fine particulate levels in indoor settings in various studies (some of which were before and after studies around local or national smokefree laws)

**Settings**	**Legally non-smoking settings (Mean PM_2.5 _in μg/m^3^)**	**Settings with smoking permitted (Mean PM_2.5 _in μg/m^3^)**
Ireland: pubs [28]	6	36
Boston, Massachusetts (USA): bars [29]	8*	179*
Austin, Texas: bars [30]	11	151
Delaware (USA): bars/restaurants (US)** [10]	11	160
**New Zealand: All pubs, bars, & restaurants in this study**	**16**	--
Malaysia: bars, nightclubs and discos [31]	22	537
Western New York (USA): bars/restaurants [7]	25	324
Thailand: bars, nightclubs and discos [31]	27	661
20 US states & Puerto Rico: hospitality venues (n = 790) [32]	28	273
Germany: bars/discos (median values) [33]	--	195/869
North West England: pubs [8]	--	286
24-country study of 932 indoor venues [4]	--	317

* Actually "PM_3.5_", though this is closely related to PM_2.5 _as described in this study.

** Based on the six bars/restaurants (and excluding the casino and pool hall).

In addition to the studies in Table 2 showing reductions in fine particulate levels after smokefree laws, there is also other evidence for hazard reduction from such laws from the United States [18-20], Norway [21-23], and Ireland [24,25].

### Study limitations

The major limitation of this study was the absence of air quality data prior to the law change to allow before/after comparisons in the same venues. Nevertheless, we were able to make comparisons with our results for "outdoor" smoking areas and with other studies in developed countries where indoor smoking is (or recently was) permitted (Table 2). The expanding data set for the "Global Air Monitoring Study" will also continue to facilitate such comparisons in the future.

This study involved sampling in only one city and one rural area of New Zealand and hence may not necessarily be representative for the rest of the country. Also, for two of the hospitality venue sampling categories, the sampling times included both the afternoon and evening and so may involve some under-estimate of typical exposure levels during busy evening periods. This is because it is more likely that people smoke outside the doorways as venues get busier in the evening (with possible spread of SHS indoors), and also more particulates may be generated by cooking and from indoor heating. To determine the air pollution contribution from these other sources, the use of additional testing equipment (eg, to detect nitrogen oxides from unflued gas heaters) may be warranted.

Finally the time of the year that this study was conducted (with relatively cold outdoor temperatures) was not conducive to the use of "outdoor" smoking areas by smokers. Hence, larger summer-time sampling of such areas is warranted in future studies. However, winter studies are best for assessing pollution from other sources such as open fires and unflued gas heaters in hospitality settings.

### Research and policy implications

A research priority is to further investigate the air quality hazard posed to customers and hospitality workers who use "outdoor" smoking areas, particularly the highly enclosed ones with walls and partial roofing. Similarly, the movement of SHS from "outdoor" smoking areas to indoor areas needs to be studied (especially in summer when windows and doors are left open and the SHS dispersion to other areas is likely to be greater). Nevertheless, a precautionary approach for such countries as New Zealand would be act now to regulate for further restrictions on the degree of enclosure allowed for "outdoor" smoking areas, and on the permitted proximity to non-smoking areas. There are many jurisdictions in the US that ban smoking in outdoor "patio" areas of hospitality venues or have large distance restrictions eg, up to 50 feet (15 m) from the entrance or exit of an establishment [26]. Similar laws for various outdoor hospitality venues operate in Singapore, and parts of Australia and Canada [27].

## Appendix

Table A1: Results for fine particulate levels (PM_2.5_) detected inside and outside various settings in this study (for 30 minute intervals exception for shorter periods in some transport settings)

**Table:** 

**Category of setting**	**Further details of site**	**Mean**(μg/m^3^)	**Median**(μg/m^3^)	**Min-imum**(μg/m^3^)	**Max-imum**(μg/m^3^)
Urban bar areas (random sample)	Bar area – A	17	18	13	24
	Bar area – B	14	15	10	22
	Bar area – C	21	20	14	33
	Bar area – D	19	18	13	32
	Bar area – E	15	13	11	37
	Bar area – F	24	24	19	32
	Bar area – G	29	30	20	42
	Bar area – H	33	30	12	56

Urban restaurants (random sample)	Restaurant – A	6	6	5	7
	Restaurant – B	17	17	16	19
	Restaurant – C	26	24	22	37
	Restaurant – D	5	5	2	7
	Restaurant – E	13	9	4	31
	Restaurant – F	13	13	6	21
	Restaurant – G	12	12	9	14
	Restaurant – H	21	23	12	28

Urban bars – more traditional (random sample)	Bar – A	9	8	4	18
	Bar – B	7	7	4	10
	Bar – C	5	5	2	7
	Bar – D	6	5	4	8
	Bar – E	4	4	3	5
	Bar – F	12	12	7	17
	Bar – G	18	18	13	25
	Bar – H	6	5	4	8
	Bar – I	26	18	9	94
	Bar – J	38	38	28	46

Rural pub – more traditional, (purposeful sample)	Pub – A	7	7	6	9
	Pub – B	7	7	4	12
	Pub – C	21	20	16	28
	Pub – D	12	12	9	18
	Pub – E	66	63	2	109
	Pub – F	15	15	11	20
	Pub – G	6	4	2	40
	Pub – H	2	2	1	5

"Outdoor" smoking areas (random sample unless otherwise stated)	Smoking area – bar 1	19	15	8	55
	Smoking area – bar 2	75	43	13	189
	Smoking area – restaurant 1	30	23	9	103
	Smoking area – restaurant 2	20	15	7	51
	Fairly enclosed smoking area (purposeful sample) – 1	65	50	20	146
	Fairly enclosed smoking area (purposeful sample) – 2	182	183	104	284

Other indoor venues that are legally smokefree (convenience sample)	Café – A	5	5	3	10
	Café – B	3	2	1	9
	Club	5	4	4	8
	Hospital site	2	2	1	4
	Public library	0	0	0	1
	Office – A	4	3	2	9
	Office – B	1	1	1	1
	Shopping centre	6	5	3	14
	Supermarket	0	0	0	3

Outside areas – not usually smokefree (convenience sample)	Roadside – A	3	3	2	5
	Roadside – B	4	4	3	6
	Park – A	13	11	5	50
	Park – B	5	4	2	12
	Walkway – A	9	8	5	15
	Walkway – B (legally smokefree)	9	9	4	15

Transport settings (convenience sample)	Train station/airport – A	2	2	1	3
	Train station/airport – B	9	9	5	14
	Bus station	15	13	12	48
	Bus trip – A	24	24	9	62
	Bus trip – B	4	4	3	4
	Bus trip – C	21	18	6	58
	Bus trip – D	20	17	10	34
	Bus trip – E	21	21	13	28
	Taxi trip	8	8	6	10
	Train trip	7	6	3	16

## Competing interests

Two of the authors (NW and RE) have previously undertaken work for the Ministry of Health and for not-for-profit organisations involved in tobacco control.

## Authors' contributions

The first two authors were involved in designing the project. Field work was undertaken by four of the authors (all except RE). NW analysed the data and prepared the first draft of the manuscript. All the authors were involved in re-drafting of the manuscript and have given final approval of the version to be published.

## Pre-publication history

The pre-publication history for this paper can be accessed here:


